# Ecosystem recharge by volcanic dust drives broad-scale variation in bird abundance

**DOI:** 10.1002/ece3.1523

**Published:** 2015-05-25

**Authors:** Tómas Grétar Gunnarsson, Ólafur Arnalds, Graham Appleton, Verónica Méndez, Jennifer A Gill

**Affiliations:** 1 University of Iceland, South Iceland Research CentreFjölheimar, Bankavegur IS-800 Selfoss and Gunnarsholt, IS-851, Hella, Iceland; 2 Faculty of Environmental Sciences, Agricultural University of IcelandHvanneyri, IS-311, Borgarnes, Iceland; 3 British Trust for Ornithology, The NunneryThetford, Norfolk, IP24 2PU, UK; 4 School of Biological Sciences, University of East Anglia, Norwich Research ParkNorwich, NR4 7TJ, UK

**Keywords:** Atmospheric dust, bird abundance, desertification, ecosystem recharge, ecosystems, global warming, shorebirds, volcanic dust

## Abstract

Across the globe, deserts and volcanic eruptions produce large volumes of atmospheric dust, and the amount of dust is predicted to increase with global warming. The effects of long-distance airborne dust inputs on ecosystem productivity are potentially far-reaching but have primarily been measured in soil and plants. Airborne dust could also drive distribution and abundance at higher trophic levels, but opportunities to explore these relationships are rare. Here we use Iceland's steep dust deposition gradients to assess the influence of dust on the distribution and abundance of internationally important ground-nesting bird populations. Surveys of the abundance of breeding birds at 729 locations throughout lowland Iceland were used to explore the influence of dust deposition on bird abundance in agricultural, dry, and wet habitats. Dust deposition had a strong positive effect on bird abundance across Iceland in dry and wet habitats, but not in agricultural land where nutrient levels are managed. The abundance of breeding waders, the dominant group of terrestrial birds in Iceland, tripled on average between the lowest and highest dust deposition classes in both wet and dry habitats. The deposition and redistribution of volcanic materials can have powerful impacts in terrestrial ecosystems and can be a major driver of the abundance of higher trophic-level organisms at broad spatial scales. The impacts of volcanic ash deposition during eruptions and subsequent redistribution of unstable volcanic materials are strong enough to override effects of underlying variation in organic matter and clay content on ecosystem fertility. Global rates of atmospheric dust deposition are likely to increase with increasing desertification and glacier retreat, and this study demonstrates that the effects on ecosystems are likely to be far-reaching, both in terms of spatial scales and ecosystem components.

## Introduction

Productivity and patterns of biodiversity within ecosystems vary greatly in space and time, with important consequences for ecosystem function and conservation strategies (Hooper et al. 2005). At different spatial scales, the level of nutrient input into ecosystems is a key driver of variation in primary productivity, species richness, and population density (Einarsson et al. 2004; Langmann et al. 2010; Sigurdsson and Magnusson 2010). Nutrient inputs can be diverse, of both anthropogenic and natural origin, and are transported into and through ecosystems by air, water, and biological cycles. In terrestrial habitats, chemical weathering, soil formation, and maintenance underpin ecosystem development and primary productivity. As soils develop and age, important nutrients can be diminished, through leaching and removal by anthropogenic land use, and eventually ecosystem fertility is reduced if nutrients are not replaced. Numerous studies show that airborne inputs, often from distant sources, are fundamental in recharging oceanic (Jickells et al. 2005; Langmann et al. 2010) and terrestrial (Chadwick et al. 1999; Field et al. 2010) ecosystems and can have long-lasting effects (Reynolds et al. 2006). For example, African dust is often cited as a major source of nutrients in the Atlantic (Prospero et al. 2002; Engelstaedter et al. 2006; Ridley et al. 2012; von Suchodoletz et al. 2013) and it also influences European terrestrial ecosystems (Lequy et al. 2012). African dust sources have also been suggested to be involved in the demise of Caribbean coral reef systems, highlighting the potential scale of the ecological effects of airborne dust (Shinn et al. 2000; Garrison et al. 2003). Although the effects of airborne inputs on nutrients and chemical properties in ecosystems are potentially far-reaching, these have primarily been measured at the level of soil or vegetation (McTainsh and Strong 2007; Field et al. 2010; Sankey et al. 2012; Eger et al. 2013) and at relatively local spatial scales.

The potential scale of dust deposition effects on ecosystems has only recently been fully appreciated (McTainsh and Strong 2007; Field et al. 2010; Arnalds and Dagsson-Waldhauserova 2014), but a lack of variation in deposition rates at scales relevant to mobile organisms at higher trophic levels, such as vertebrates, makes exploration of these processes difficult. In addition, anthropogenic impacts on land, such as widespread application of fertilizers and habitat degradation, are likely to mask effects of dust inputs on the abundance of species at higher trophic levels. It is likely that global dust emissions will increase in the near future, due to increasing desertification resulting from human activities and climate change (Field et al. 2010). Glacial retreat is also likely to contribute to atmospheric dust through exposing areas in which volcanic dust has previously been deposited (Gisladottir et al. 2005; Bullard 2013). Understanding the ecosystem-level influence of dust emissions is therefore of growing importance.

Globally, more than 1500 volcanoes have been active over the last 10,000 years (Siebert et al. 2010). Volcanic eruptions are frequent events, which is exemplified by the regular (every ∽1.25 months) occurrence of ash clouds over the North Pacific (Webley et al. 2012). Many volcanoes emit vast amounts of deposits that traverse the globe to varying extents and directly influence ecosystems particularly close to eruption sites (Nriagu 1980). Although the immediate catastrophic effects of eruptions on ecosystems adjacent to volcanoes have been well-documented (McTainsh and Strong 2007; Arnalds 2013; Eger et al. 2013), their effects on ecosystems at broader spatial scales are poorly known. Unstable volcanic deposits can become a source of widespread eolian redistribution of volcanic materials (Arnalds 2013), as exemplified by the aftermath of the 1991 Hudson eruption in Chile (Wilson et al. 2011) and the 2010 Eyjafjallajökull eruption in Iceland (Arnalds et al. 2013).

The glacial margins, floodplains, and desert areas of Iceland consist of volcanogenic materials and are among the most active dust sources on Earth (Arnalds et al. 2014; Arnalds 2010; Prospero et al. 2012; Dagsson-Waldhauserova et al. 2013). The volcanic glasses generated from these dust sources have a controlling effect on Icelandic soils (Arnalds 2008; Arnalds and Oskarsson 2009), as they largely determine the organic and clay content of the soils. These volcanogenic basaltic dust materials are poorly crystalline, often porous, with many broken edges. This results in rapid weathering, which rates among the most rapid on Earth, with chemical denudation rates up to 150 t km^−2^ year^−1^ (see Arnalds 2015; Arnalds et al. 2014). The weathering releases a suite of important elements, such as Ca, K, and Mg, which enhance fertility by maintaining favorable pH and cations which are important for nutrition. This is not the case with more uniform silica-rich dust that is characteristic of most global dust sources. This reactivity and the highly spatially variable deposition rates (from <50 to >1000 g m^−2^ year^−1^; Arnalds 2010) provide a unique opportunity to assess the effects of nutrient recharge by volcanic dust on ecosystem processes at broad spatial scales.

The Icelandic terrestrial vertebrate fauna is characterized by low species richness (one native mammal, no reptiles or amphibians, and ca. 80 regularly breeding bird species) but by very large bird populations. In particular, very large populations of ground-nesting waders (Charadrii), which are open-habitat specialists, occur in internationally important (1% of a population: Birdlife International 2004) numbers across lowland Iceland (Gunnarsson et al. 2006). These species are mostly secondary consumers which feed on invertebrates. Surveys of lowland-breeding waders throughout Iceland have shown a very high level of broad-scale spatial variation in abundance, despite comparable availability of suitable habitat types (Gunnarsson et al. 2006), but the cause of this variation is not known. In order to assess the influence of volcanic dust deposition rates on ecosystems and their higher trophic-level species, we explore the links between broad-scale variation in volcanic dust deposition rates (Arnalds 2010) and breeding bird abundance (Gunnarsson et al. 2006), within and between habitats subject to differing levels of anthropogenic influence in lowland Iceland.

## Materials and Methods

### Study area

Iceland is a volcanic island of ∽100,000 km^2^ located in the North Atlantic Ocean, between 63°23′N and 66°32′N latitudes. It has a relatively mild climate for its northern location owing to warm ocean currents (Einarsson 1984), with cool summers (∽10°C average in lowlands) but mild winters (∽0°C average in lowlands, http://www.vedur.is). Annual precipitation ranges from ∽300 mm in northeast Iceland to >2000 mm in mountains in the south. Iceland is mountainous with about 25% of the land located below 200 m elevation and is part of the Mid-Atlantic Rift Zone with an active volcanic belt running through the country. The volcanism is amplified by an active volcanic hotspot under the country, resulting in frequent volcanic eruptions, approximately one every 4–5 years (Thordarson and Höskuldsson 2008). About 10% of Iceland is covered with glaciers, which cap many active volcanoes (Jakobsson and Gudmundsson 2008), and subglacial eruptions can result in flooding and the release of new unstable volcanic materials at glacial margins.

Sandy areas cover about 22,000 km^2^ of Iceland, but the sandy materials consist mainly of basaltic to andesitic volcanic glass with some basaltic rock fragments (Arnalds et al. 2001; Baratoux et al. 2011). These sandy areas have extremely unstable surfaces and are subjected to intense wind erosion and dust formation (Arnalds 2010; Arnalds et al. 2012; Prospero et al. 2012; Thorarinsdottir and Arnalds 2012). The parent material of Icelandic soils is mainly volcanic glass, deposited during eruptions and as eolian materials during frequent dust events originating from these dust sources.

As the soils develop, allophane, imogolite, and ferrihydrite clay minerals form, together with organic carbon accumulation, which are generally inversely related to the dust accumulation. Arnalds (2008, 2010) and Arnalds and Oskarsson (2009) described the soil properties of Icelandic soils in relation to the dust inputs. In wetlands, organic content is highest in surface horizons furthest away from the eolian dust sources with >20% C in surface horizons but 12–20% C at intermediate levels of dust deposition. Wetlands closer to eolian dust sources have lower organic content (<12% C; Gleyic Andosols), and where dust accumulation is high, the content is often 0.5–4% in surface horizons, mainly dependent on the amount of dust deposition. Soil reaction (pH) in wetlands distant from dust sources is relatively low (pH 4–5) compared to wetlands close to dust sources (pH 5.5–7). Organic wetlands (Histosols and Histic Andosols) have low clay content (2–6%). The dryland soils have a pH gradient from ∽7 near major dust sources to ∽5 in areas far from dust sources. The clay and organic content of soils are generally considered among the key factors controlling nutrient availability and, hence, the overall fertility of these ecosystems.

The effects of dust input are likely to vary between distinct habitats, and we considered the effects of dust separately for agricultural habitats (95% hayfields and 5% crops, potatoes, and kale), dry habitats (heathlands and grasslands), and wetland habitats (marshes and bogs). Agricultural habitats receive manure and artificial fertilizers up to the desired level for farming (mainly hay-making, see Helgadóttir et al. 2013), which could mask the effects of volcanic dust. Wetlands and dry areas were treated separately because wetland habitats are inherently more fertile systems, due to higher organic contents and greater water availability than the dry habitats, and because the generally lower pH in wetlands may also bring about more rapid weathering and nutrient release from the basaltic volcanic glass. Icelandic wetlands cover 7–10,000 km^2^ (7–10%), of Iceland, and are mainly found in lowland areas below 400 m elevation (Arnalds 2011). The Icelandic wetland soils have high mineral content and relatively low organic carbon content (generally 2–20%), which separates them from other high latitude wetland soils (Arnalds 2008). These soils classify mostly as Aquands or Aquic Andosols (US Soil Taxonomy and WRB classification systems, respectively), with so-called andic soil properties dominating, while more organic Histosols are found in areas receiving the least amount of dust inputs (Arnalds and Oskarsson 2009).

### Spatial variation in dust deposition rates

**Figure 1:**
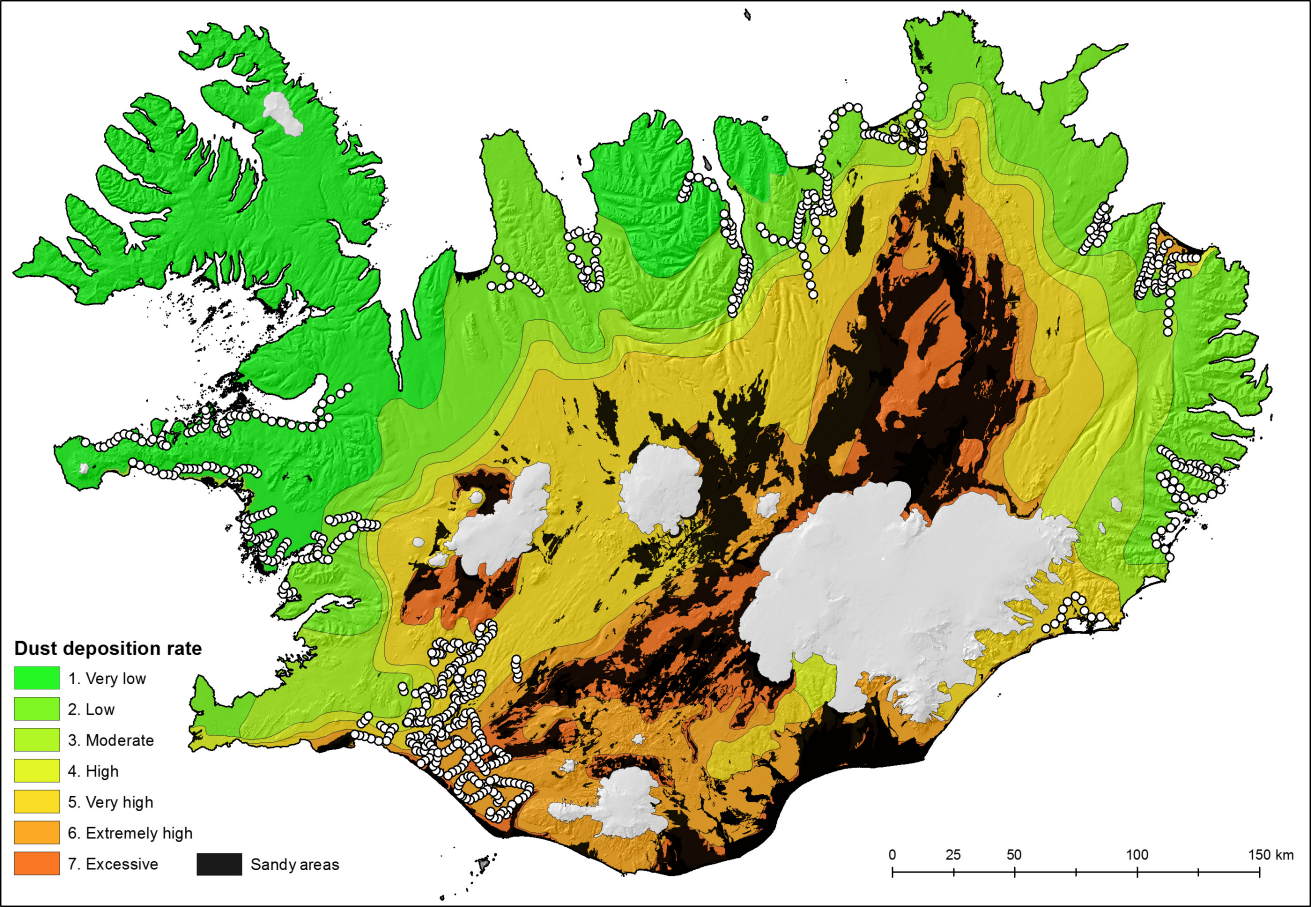
Map of Iceland showing spatial variation in dust deposition rates, with higher rates typically closer to the North Atlantic Rift Zone running from southwest to northeast, and the location of the bird survey locations (white circles). Sandy areas (shown in black) were not surveyed. White areas are glaciers.

The average annual dust distribution in Iceland was mapped by Arnalds (2010) with parameters including (1) directly measured soil thickening rates using profile descriptions employing tephra (ash) layers of known age from published sources and from the Agricultural University of Iceland soil database; (2) the position of the main dust source areas, which include primary dust plume areas and larger unstable sandy areas; and (3) prevailing dry winds (direction and strength) for each of the dust source areas; (4) key landscape features such as mountains. These results have also been verified using satellite imagery showing the major dust sources during high intensity storms and the extent of the plumes, often several hundred kilometers for the sources (Arnalds 2010; Arnalds et al. 2014). Deposition rates are divided into seven classes with one being the lowest deposition rates and seven the highest (Fig.1), and the average number of bird survey patches within each dust deposition class was 104 (standard deviation = 67, range 45–249, Table1). The average annual number of dust days (days when dust is recorded at one of the stations operated by the Icelandic Meterological Office) in Iceland is >130 days (Dagsson-Waldhauserova et al. 2014a,b). Dust deposition in Iceland exceeds 1000 g m^−2^ year^−1^ closest to the most active dust sources and is >500 g m^−2^ year^−1^ within areas of widespread sandy deserts, but the deposition rates drop rapidly with distance from the sandy deserts, with more coarse grains settling out from the atmosphere, while smaller particles are carried over longer distances, often exceeding 500 km (see Arnalds 2010; Dagsson-Waldhauserova et al. 2013). There is a 100-fold drop in deposition rates from areas close to dust plume sources to the areas furthest away from the sources. The seven deposition classes represent a wide range in deposition (Table1). The eolian deposition rates have controlling influences on soil properties and are used for classifying Icelandic soils (Arnalds and Oskarsson 2009). Both carbon content and pH in the top 30 cm of soils decline markedly with increasing dust deposition (Table1), and soil texture becomes finer with distance from the most active eolian sources. The values for the map categories are given as a range for dust deposition. The deposition rates illustrated on the map have a close relationship with iron content measured in mosses which is primarily wind deposited (Magnússon 2013). It should be noted that the total dust emissions that lead to these dust deposition averages are similar to total dust generation calculated by an independent method based on the annual number of storms in Iceland, their severity and a dust generation model (Arnalds et al. 2014). Differences between categories on the map are quite large, hence the 100× decrease in dust deposition from category 7 to 1. There are broad variations within each category, determined by landscape features that affect wind directions and speeds. There is also broad temporal variation dependent on such factors as changes in dust sources over time, climate variations which cause short- and long-term changes in dust activity (Dagsson-Waldhauserova et al. 2014a,b) and temporary inputs or “spikes” by volcanic events. However, the influence of the gradual dust input will last for a long time, determined by weathering rates. Factors affecting pH and other soil properties are not likely to be sensitive to differences smaller than the differences between categories in deposition rates (e.g., 10–50%). In addition, lateral movement of weathering products with soil water can also be expected, especially in wetlands, reducing site differences in water chemistry. It should, however, be noted, that the relationship is tested against the dust categories, but not numerical values for dust deposition at each site per se, which is impossible to derive with more accuracy than is given by the range for each category.

**Table 1:** Typical values for carbon and pH in the top 30 cm of soil and the dominant soil colloid constituents

Dust deposition category	Number of bird survey patches	g m^−2^ year^−1^	Wetlands % C	pH	Drylands % C	pH	Dominant soil colloids
1 Very low	114	<50	>30	4–4.5	10–15	5–5.5	MHC
2 Low	249	50–100	20–30	4–4.5	8–12	5.5–6	MHC
3 Moderate	45	100–150	15–20	4.5–5	6–8	5.5–6	MHC + A F
4 High	80	150–250	10–15	5–5.5	4–6	6–6.5	A F + MHC
5 Very high	83	250–500	5–10	5.5–6	2–5	6–6.5	A F + CHC
6 Extremely high	86	500–1000	1–5	6–6.5	1–3	6.5–7	A F (low)
7 Excessive	72	>1000	<1	6–7	0.5–2	7–7.5	A F (very low)

From Agricultural University of Iceland Soil Database (see Arnalds et al. 2014; chapters 6 and 8).

MHC, metal–humus complexes; A, allophane; F, ferrihydrite; CHC, clay–humus complexes.

### Spatial variation in bird abundance

Gunnarsson et al. (2006) reported the results of a survey of densities of breeding birds in lowland Iceland which took place between 2001 and 2003. In total, 758 survey patches (mean size = 2.1 ha ± 1.3 SD, patch size ranged from 1.63 to 2.46 ha per dust category) located every 2 km (or occasionally every 4 km) along roads and tracks throughout lowland Iceland were visited once, and the numbers of breeding birds and habitat type and structure were recorded at each patch. During each stop, a GPS point was recorded and, from that location, the number of birds on the adjacent patch of homogenous habitat was counted and the size of the patch was estimated. Counts at each stop lasted less than 5 min in all cases. Visits were conducted in the last 2 weeks of May each year, when the activity of breeding birds is at a maximum (Johannesdottir et al. 2014). Most of the interior of Iceland is a barren highland plateau, and all surveys were carried out in coastal lowland basins below 200 m a.s., so broad-scale variation in topography is negligible. All the habitats included in this analysis have short vegetation and unobscured views, and the bird species using these habitats are highly conspicuous during the breeding season. Comparisons of the detectability of individual wader species between these common habitat types have found no differences between species (Gunnarsson et al. 2012; Johannesdottir et al. 2014), and the habitat types used here are common in all parts of the country (Gunnarsson et al. 2006). The climate of Iceland is mild and oceanic and does generally not vary greatly between parts of the country or systematically with dust deposition but much of the variation in temperature is on a coast-inland gradient within lowland basins (Einarsson 1984). We used a GIS analysis to relate climate variables (multidecadal means of temperature and precipitation) to bird abundance, which showed that the relationship between bird abundance and climate on survey points was absent or very weak (Appendix S1). For the purpose of this study, survey patches in towns, lakes, lava-fields, and forestry were excluded, leaving 729 patches available for analysis. The remaining habitats were grouped into wet (marshes, dwarf birch bogs, and all locations containing pools or bodies of open water; *n* = 152 patches), dry (heathland, riverplains, dry grasslands, and unvegetated areas; *n* = 383), and agriculture (*n* = 194).

### Data analysis

We modeled the total number of breeding waders (Charadrii), at survey patches as a function of the level of local volcanic dust deposition (Fig.1), using a generalized linear model. Overdispersion of the data meant that a negative binomial error distribution and a log link function provided the best model fit. We included the size of the survey patch (ha) as a covariate in the models, habitat type (agriculture, wet, dry) and dust deposition class as fixed factors, and a dust deposition x habitat type interaction term to assess whether the effects of dust vary among habitats. We then constructed separate models for each of the seven wader species (oystercatcher *Haematopus ostralegus*, golden plover *Pluvialis apricaria*, black-tailed godwit *Limosa limosa*, whimbrel *Numenius phaeopus*, redshank *Tringa totanus*, snipe *Gallinago gallinago*, and dunlin *Calidris alpina*), using generalized linear models with a Poisson distribution and a log link function, and the same covariates, fixed factors, and interactions as above. Poisson error distribution fitted the individual species data better than the negative binomial which was fitted to the combined species model. In order to explore whether survey patches were clustered in respect to bird abundance and to check that we were not violating independent error assumptions of generalized linear models, we assessed the extent to which the residuals of each model were spatially autocorrelated by calculating Moran's I on the residuals of each model using the Moran I function in the R package *ape* at 5 km lag distance class (Paradis et al. 2004).

## Results

### Influence of dust deposition on the distribution and abundance of birds

**Figure 2:**
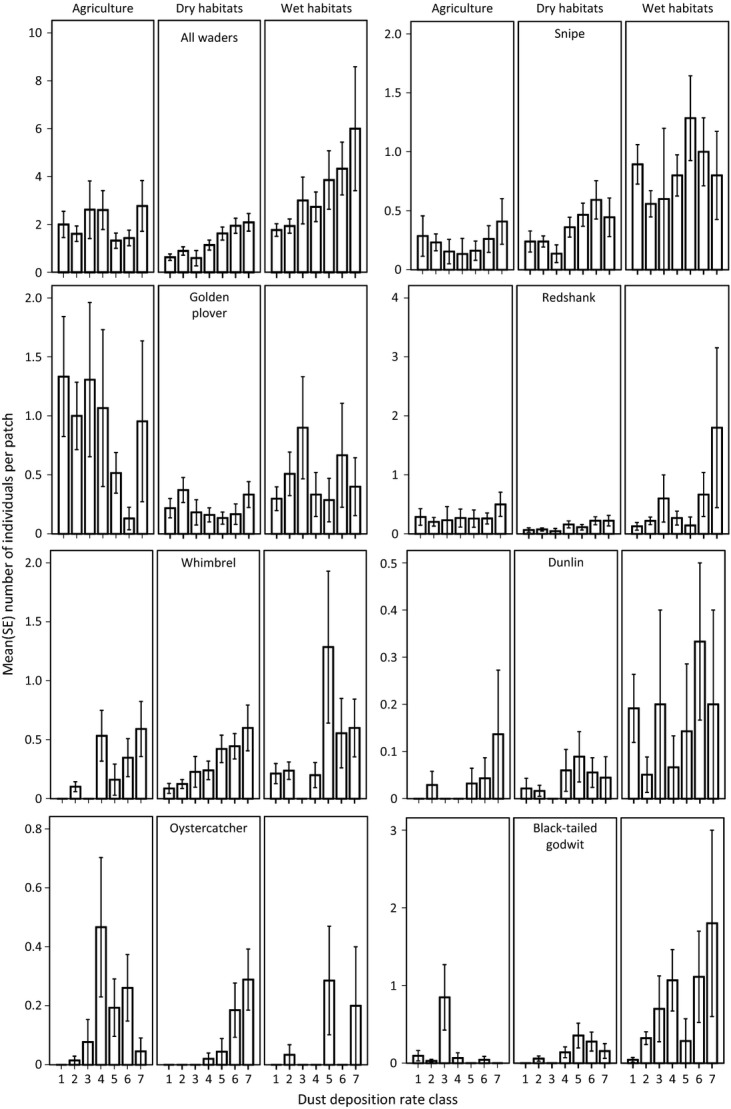
The variation in mean (±SE) number of individuals of seven species of waders, combined and by individual species, in agricultural, dry, and wet habitats in areas of lowland Iceland with differing levels of dust deposition rate (7 = highest deposition rate).

The mean number of all waders recorded at survey patches differed significantly among habitat types (Table2) and was highest on wet habitats (mean = 2.4 waders/patch ± 2.6 SD), intermediate on agricultural land (1.8 ± 2.9 SD), and lowest on dry habitats (1.2 ± 1.9 SD) (Fig.2). Within both wet and dry habitats, but not in agricultural land, bird abundance was significantly higher in areas with higher levels of dust deposition (Table2, Fig.2). Within both dry and wet habitats, the total abundance of breeding waders tripled, on average, between the lowest and highest deposition classes (Table2, Fig.2). Spatial autocorrelation in the residuals of the model including all species was significant but weak (Moran's I estimate is very close to 0) (Table3).

**Table 2:** Results of generalized linear models of the variation in the number of individuals of seven common species (combined and by species) of waders in areas of lowland Iceland in relation to levels of dust deposition, habitat type (agricultural, dry, or wetland), and survey patch size

	All waders			Snipe		
	Wald LR	df	*P*	Wald LR	df	*P*
Dust deposition class	20.505	6	0.002	9.876	6	0.1299
Patch size	30.703	1	<0.0001	32.275	1	<0.0001
Habitat type	33.359	2	<0.0001	30.848	2	<0.0001
Dust × habitat	18.701	12	0.096	7.918	12	0.7915
Overall model fit (LR Chi-Square)	127.695	21	<0.0001	115.618	21	<0.0001
Deviance/df	1.043			1.039		

	Golden Plover			Redshank		
	Wald LR	df	*P*	Wald LR	df	*P*
Dust deposition class	89.660	6	0.004	39.341	6	<0.0001
Patch size	19.142	1	0.002	0.061	1	0.805
Habitat type	9.268	2	<0.0001	19.274	2	<0.0001
Dust × habitat	24.852	12	0.016	15.816	12	0.200
Overall model fit (LR chi-Square)	168.536	21	<0.0001	70.561	21	<0.0001
Deviance/DF	1.583			0.759		

	Whimbrel			Dunlin		
	Wald LR	df	*P*	Wald LR	df	*P*
Dust deposition class	34.864	6	<0.0001	6.566	6	0.363
Patch size	21.347	1	<0.0001	8.877	1	0.003
Habitat type	1.921	2	0.383	4.358	2	0.113
Dust × habitat	12.085	9	0.209	5.681	8	0.683
Overall model fit (LR chi-Square)	123.414	21	<0.0001	44.544	21	<0.0001
Deviance/DF	0.846			0.317		

	Oystercatcher			Black-tailed Godwit		
	Wald LR	df	*P*	Wald LR	df	*P*
Dust deposition class	16.023	5	0.007	47.211	6	<0.0001
Patch size	5.134	1	0.023	23.983	1	<0.0001
Habitat type	4.513	2	0.105	20.467	2	<0.0001
Dust × habitat	13.575	5	0.019	33.724	8	<0.0001
Overall model fit (LR chi-Square)	108.104	21	<0.0001	218.777	21	<0.0001
Deviance/DF	0.318			0.657		

**Table 3:** Moran's autocorrelation index for the residuals of models for all waders combined and for individual species at 5 km lag distance

Species	Moran's I	*P* value
All waders combined	0.0097	0.038
Oystercatcher	0.0077	0.083
Golden plover	−0.0012	0.978
Whimbrel	0.0018	0.557
Black-tailed Godwit	0.0050	0.232
Snipe	0.0366	<0.001
Redshank	0.0008	0.689
Dunlin	−0.0016	0.966

### Influence of dust deposition on abundance of individual species

The abundance of five of the seven common wader species varied significantly in relation to dust deposition rates, despite the abundance of each species varying greatly between habitats (Table2, Fig.2). Of the five species that are most abundant on wetland habitats (snipe, redshank, whimbrel, dunlin, black-tailed godwit), only snipe did not show an increase in abundance with dust deposition (Fig.2). The two species that are more abundant on agricultural habitats (golden plover and oystercatcher) had significantly differing effects of dust deposition between habitats (Table2, Fig.2), with no clear effect of dust within agricultural habitats, but oystercatchers increased in abundance with dust levels in wet and dry habitats (Fig.2). Black-tailed godwits also had significantly differing effects of dust between habitats, primarily because their abundance increased markedly with dust levels on wetlands, but they occur too infrequently on agricultural and dry habitats to identify any such response (Fig.2). Spatial autocorrelation was absent from the residuals of these models, with the exception of snipe, where residuals were significantly but weakly positively autocorrelated (Table3).

## Discussion

Across Iceland, volcanic eruptions and vast sandy areas periodically release high volumes of dust of volcanic origin over much of the country. This dust is largely basaltic, and subject to rapid weathering and release of a suite of cations (Gislason 2008) that are important for terrestrial ecosystem function (Arnalds 2010). The abundance of waders, which are the dominant group of terrestrial birds in Iceland, was strongly related to variation in the rate of this volcanic dust deposition and tripled on average between the lowest and highest deposition classes in wet and dry habitats. The increased bird abundance in areas of high dust deposition was particularly strong in wetlands and was also pronounced in dry habitats. Effects of dust deposition were not detected in agricultural lands, which may be because fertilizers, and land use can affect nutrients levels and mask natural variation in agricultural lands. These bird species are typically secondary consumers that primarily consume invertebrates, and their direct response to variation in dust deposition rates is likely to reflect transcending, bottom-up effects of dust deposition on bird abundance. This suggests that eolian and volcanic inputs to Icelandic terrestrial ecosystems are fundamental to their productivity. Organic matter and clay contents are commonly cited as key components of soil fertility (Brady and Weil 2013), but these factors show an inverse relationship with dust accumulation in Iceland (Arnalds 2010), contrary to the abundance trends exhibited by most of the bird species. This indicates that the fertility of the basaltic volcanogenic dust and rapid deposition rates override the importance of the organic matter and clay content when it comes to the redistribution of nutrients through nutrient cycling and buffering of soil pH. The pH levels of soils can directly influence the invertebrate food resources of birds, such as earthworms which perform poorly in acidic soils (Curry 2004), and are a key resource for waders (Ausden et al. 2001; Verhulst et al. 2007). The strong positive response of waders to higher deposition rates, particularly the larger Scolopacid waders (Fig.2), may therefore reflect aggregation in areas with abundant food resources. Another possible point of importance is that metal–humus complexes (Table1) dominate Andosol systems at low pH, but allophane formation is inhibited at pH < 5. This complexity may immobilize the organic matter and thus have a negative effect on energy availability for nutrient cycling in the soil. A few species showed inconclusive relationships with dust deposition. The reasons for this may be either biological or an attribute of the dataset but these patterns would have to be addressed with a finer-scale study design.

Current patterns of abundance in common land birds in Iceland are largely the product of events following the settlement of humans in the last 1100 years. Lowland Iceland was mostly covered in birch (*Betula pubescens*) woodland at the time of settlement (McGovern et al. 2007), a habitat unfavorable for most of the ground-nesting bird species that are now common and which prefer open habitats (Gunnarsson et al. 2006). A major decline in the distribution of birch began around the year 1200, and by 1500 only an estimated 5% remained (Gathorne-Hardy et al. 2009). This landscape-scale land degradation was the product of an interaction of unsustainable clear-cutting of woodlands, overgrazing volcanic activity, and climatic change (Aradóttir and Arnalds 2001). Since this time, much of the lowland area of Iceland has become available as suitable breeding habitat for many ground-nesting birds, which now occur in internationally important numbers in Iceland. The strong influence of the highly variable dust deposition rates across Iceland on the current spatial variation in bird abundance is most likely driven by impacts of dust on habitat quality, demographic rates, and dispersal patterns (Gunnarsson et al. 2005; Gunnarsson et al. 2012).

Temporal variation in deposition rates in recent centuries is likely to have followed both increased soil erosion, especially during the middle ages (Oskarsson et al. 2004), and increased dust inputs from major sandy dust source areas. Many of the desert source areas are likely to have been present prior to human settlement, at glacial margins and glaciogenic floodplains, but advances of glaciers during the last millenium and especially glacial retreat over the past 100 years (Björnsson and Palsson 2008) have caused increased dust deposition from these sources. In that respect, the highly variable deposition rates which we have linked to bird abundance are likely to have originated in the settlement period, which is supported by evidence of 4–10 times slower soil thickening rates prior to the settlement (Thorarinsson 1961; Arnalds 2010). This inference does not exclude a potentially large additional effect of deposition further back in Iceland's history, as deposition effects on ecosystems can last for millennia (Reynolds et al. 2006), particularly in warm periods where glaciers were retreating, leaving vast open glacial margins. Due to volcanic activity along the Rift Zone, deposition rates of ash are always likely to have been highest on a SW-NE axis.

Although studies reporting a direct relationship between volcanic dust deposition and broad-scale abundance of birds have seemingly not been previously reported, soil type has frequently been related to habitat quality and abundance. A rare link with broad-scale bedrock type was reported by Kålås et al. (1997) who found that the locations of great snipe (*Gallinago media*) leks throughout Scandinavia were strongly associated with base-rich bedrock, soils with high pH and high earthworm biomass. This example constitutes a comparable ecological pattern to our study but a distinct geochemical process. Similarly Wilson et al. (2005) found that nightingale (*Luscinia megarhynchos*) territories in East England were more likely to be on humus-rich soils than not. Studies like these hint at the important long-term effects of geology on bird abundance, operating through food chains. The anthropogenic impacts masking the natural variation in geology are probably much greater in more densely populated countries with more intensive agriculture than they are in Iceland, making the effects on birds harder to identify.

The effects of dust on global ecosystems have been recognized, with dust both affecting nutrient levels and air pollution which changes albedo (reflection coefficient) of snow and glaciers (Field et al. 2010). Airborne volcanic materials deposited during volcanic eruptions are considered important on a global scale as they are continuously adding fresh material to terrestrial systems (Nriagu 1980) and oceans (Duggen et al. 2010; Olgun et al. 2011; Ayris and Delmelle 2012). It has been argued that rejuvenation with nutrient rich materials is essential to prevent ecosystem retrogression (Pelzer et al. 2010), and accumulation of volcanic dust can increase the fertility of old tropical soils (Vitousek et al. 2003). Our findings show that not only are the impacts of volcanic eruptions important in this context but also the rate of redistribution of volcanic materials as dust. This redistribution can have a strong enough impact on terrestrial ecosystems to be directly detected in secondary consumers at broad spatial scales.

Icelandic glaciers are retreating due to the warming of the atmosphere (Björnsson and Palsson 2008). Most of the major dust sources are located at glacial margins and floodplains. It is therefore likely that volcanic dust emissions will increase in the future, which has a negative effect on air quality in the country, but potentially increases primary productivity with consequences for animal abundance. Global warming does also cause glacier retreat and increased desertification elsewhere, so it is likely that the volume and redistribution of atmospheric dust will increase in near future (Field et al. 2010; Bullard 2013). Our study shows that the impacts on ecosystems have the potential to be far-reaching both spatially and across food webs.

## Supporting Information

Appendix S1 Association between bird abundance and climate variables.
