# Supplementary material for: Ecosystem recharge by volcanic dust drives broad-scale variation in bird abundance
Source: Ecol Evol. 2015 May 25;5(12):2386–96. doi: 10.1002/ece3.1523 (PMC4475371; doi:10.1002/ece3.1523)
Supplement: Appendix S1 — Association between bird abundance and climate variables. [file ece30005-2386-sd1.docx]

**Appendix S1:** Association between bird abundance and climate variables

**Supplementary material with the paper:**

Gunnarsson, T.G., Arnalds, O., Appleton, G., Méndez, V. & Gill, J.A. 2015. Ecosystem recharge by volcanic dust drives broad-scale variation in bird abundance. Ecology and Evolution.

As the abundance of breeding waders can also potentially be influenced by local climatic conditions it was necessary to also explore the association between bird abundance and local weather metrics to ensure that any apparent effects of dust deposition were not confounded by climatic conditions. Data on weather conditions across Iceland were obtained from the Icelandic Meteorological Office ([www.vedur.is](http://www.vedur.is)). Using Geographical Information Systems (ArcGIS 10.1), layers of mean annual temperature (years 1961-2006) and precipitation (years 1971-2000) were superimposed on dust distribution to obtain estimates of each weather variable for each bird sampling patch (see dust distribution on Fig. 1 in main document). Pearson correlations of the strength of the association between numbers of birds on each patch and temperature and precipitation were very weak for both weather variables (Table 1).The reported association between bird abundance and dust deposition rates are therefore unlikely to be confounded by variation in climatic conditions.

| **Table A1. Pearson correlation coefficients of the association between mean annual temperature and preciptation and bird density on lowland patches throughout Iceland.** | | |
| --- | --- | --- |
|  | Temperature | Precipitation |
| All waders combined | 0.051 | 0.092 |
| Oystercatcher | 0.151* | 0.238* |
| Golden plover | -0.022 | -0.028 |
| Whimbrel | 0.136 | 0.165* |
| Black-tailed Godwit | -0.008 | -0.018 |
| Snipe | -0.037 | 0.009 |
| Redshank | 0.039 | 0.070 |
| Dunlin | 0.047 | 0.068 |
| *p<0.05 |  |  |
